# Study on the propagation characteristics of methane-air explosion under the promotion of crushed gangue

**DOI:** 10.1038/s41598-024-51955-2

**Published:** 2024-01-24

**Authors:** Zhenqi Liu, Qiu Zhong, Yansen Lu

**Affiliations:** 1 Jiangsu Key Laboratory of Environmental Impact and Structural Safety in Engineering, China University of Mining and Technology, No1, Daxue Road, Xuzhou, Jiangsu 221116 People’s Republic of China; 2 Key Laboratory of Gas and Fire Control for Coal Mines, Ministry of Education, China University of Mining and Technology, Xuzhou, 221116 China; 3 School of Safety Engineering, China University of Mining and Technology, Xuzhou, China

**Keywords:** Fossil fuels, Energy infrastructure

## Abstract

Methane-air explosion is one of the major disasters in industrial process. The explosion strength could be influenced by the crushed coal gangue, which is widely distributed in coal mine gob and roadway. To understand the influence of the coal gangue on gas explosion, an experimental system with a 0.2 × 0.2 × 3.0 m^3^ pipeline was designed and explosion experiments of coal gangue with 5 blockage length-diameter ratios (ratio of axial blockage length to pipeline equivalent diameter) were carried out. The results show that coal gangue can cause significant disturbances to the flame front, resulting in a violent acceleration of the explosion flame. The overpressure ratio presents a negative exponential function distribution with the blockage length-diameter ratio. The influence range increases with the blockage length-diameter ratio under the condition of rich fuel, and reaches the maximum when equivalent ratio is 1.237. The explosion intensity is more sensitive to the blockage length-diameter ratio for the equivalent ratio equals 1.0 and 1.237. The formation of high-intensity explosion should be avoided by controlling the accumulation state of the overlying rock in coal mining.

## Introduction

The explosion of combustible gas is characterized by short occurrence time, high damage intensity and wide destruction range. During the explosion process, turbulence forms in the mixed gas owing to an obstacle and leads to further expansion of disaster intensity^1–6^. The obstacles are key factors affecting the explosion intensity^7–10^. When a gas explosion occurs in a gob, the flames can rapidly spread to the working area, during which the crushed gangue in the gob may disturb the flame surface and increase the combustion intensity^11–13^. Accidents such as the Jinshangou Coal Mine in 2016, the Ermugou Coal Mine in 2019, and the UBB Coal Mine (US) in 2010, all of which had caused large number casualties.

The accelerating effect of obstacles on flames has received extensive research attention in recent years^5,14–16^. Yu et al.^14^ experimentally studied the influence of a single hollow obstacle on the characteristics of methane-air explosions and found that fewer sides of the hollow shape led to a stronger acceleration effect. Wang et al.^17^ studied the effect of plate and solid obstacles on explosion flames and showed that abrupt edges or irregular shapes of obstacles can promote flame propagation. Di Sarli et al.^18^ found that a single obstacle with a larger axial length is more likely to cause higher explosion pressure. Mohamed^19^, Sun Song^8^, Yanchao Li et al.^20^ found that high turbulence intensity can significantly increase the degree of wrinkles on the flame surface and the peak overpressure. Zhenmin Luo^21^, Litao Liu et al.^22^ found that if methane premixed gases were mixed with other flammable gases, such as hydrogen, this could lead to higher intensity explosive consequences, and the overpressure has a pronounced oscillatory character.

For the explosions in gob, the confined space has a special geometric structure-the blocked state formed by coal gangue, which could disturb the explosion propagation state^2^. The geological conditions and mining techniques bring different accumulation states of the crushed gangue in gob (i.e., the length of the flame disturbed by the blockage). Fig et al.^12^ investigated the influence of crushed rocks of a single blockage ratio and blockage length on the flame propagation velocity compared with no-obstacle conditions, and found that rock masses impose a significant acceleration effect on flame propagation. Zhenqi Liu^23^, Shangyong Zhou, et al.^24^ found that the porous structure such as the crushed rock and mesh aluminium alloy have the dual effect of promoting and suppressing an explosion. Similar researches such as Ciccarelli et al.^25,26^, they found that layered ceramic spheres and their rough surfaces have a strong promoting effect on flame propagation. Moreover, the length of the unobstructed gap has also been shown to have a significant impact on the flame structure and explosion intensity.

In summary, the accumulation of coal gangue exerts a certain degree of influence on the propagation velocity of explosion flames. However, for accumulation structures formed by crushed coal gangue, the influence of blockage parameters, especially the axial blocking length, on the methane-air explosion intensity remains unclear. An explicit understanding of the gas explosion process in the gob of coal mines is therefore lacking. To address this issue, an experimental explosion system was designed in this study to investigate the variation of methane-air explosion characteristics under the crushed rock with different blockage lengths.

## Experimental setup and process

**Figure 1:**
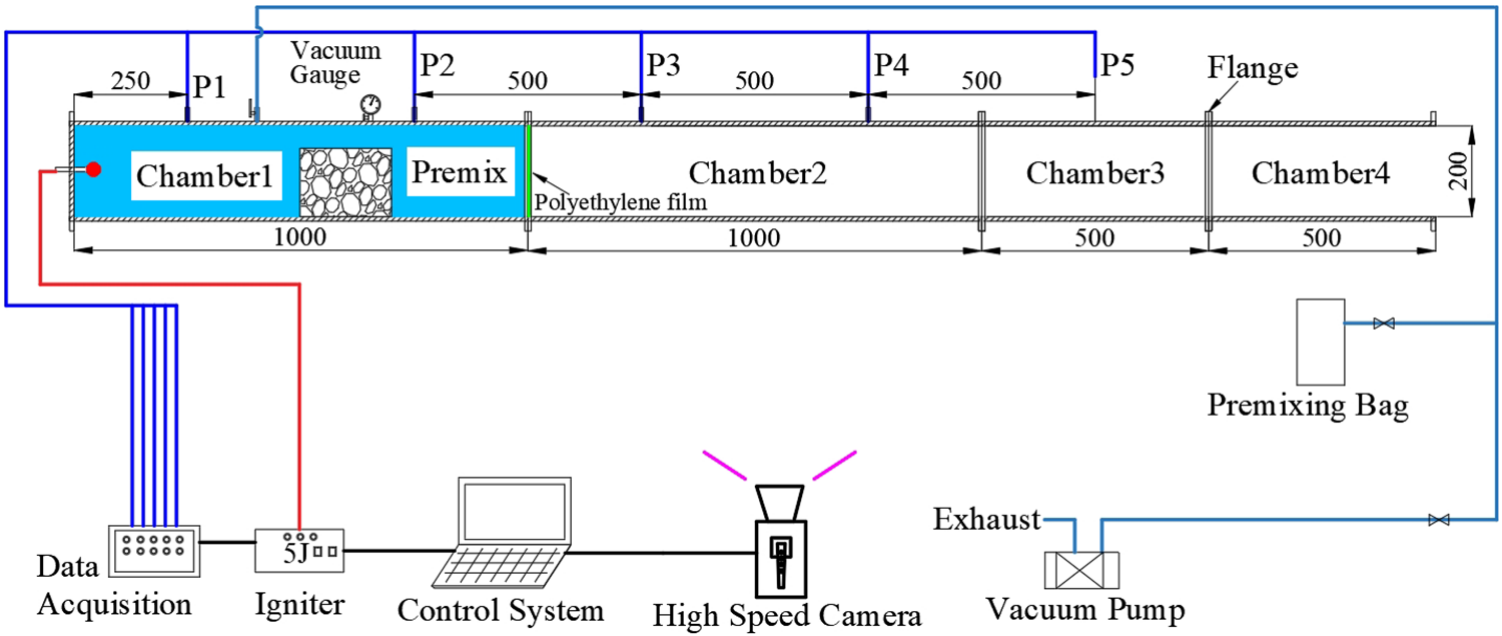
Schematic diagram of the pipeline experiment system.

The experimental system mainly includes a square pipeline with 4 quartz glass windows, 5 pressure sensors, a data collector, a high-energy igniter, a high-speed camera, and a control terminal, as shown in Fig. 1. The pipeline includes four chambers, and the internal cross-section is 0.2 × 0.2 m with a total length of 3.0 m. The volume of chamber 1 is 43 L for filling the pre-mixed gas. The ignition source is located at the closed end of chamber 1 and the chamber 4 is open to atmosphere. The height of the quartz glass window is 200 mm, which is used to observe the flame shape at the full height of the pipeline. Five pressure measurement points were arranged in the experimental pipeline. Point 1 is 0.25 m from the ignition source and the remaining points are arranged at intervals of 0.5 m. Five 2300V3 pressure sensors (Dytran Instrument Company, USA) were used with a data acquisition frequency of 250 kSa/s. The ignition energy was 5 J. The resolution of the high-speed camera was 1280 × 800, and the frame rate was 2000 fps. Pure methane (99.9%) and air were used to prepare the mixed gas with equivalent ratios (φ) of 0.772, 1.0, 1.237, and 1.486.

**Figure 2:**
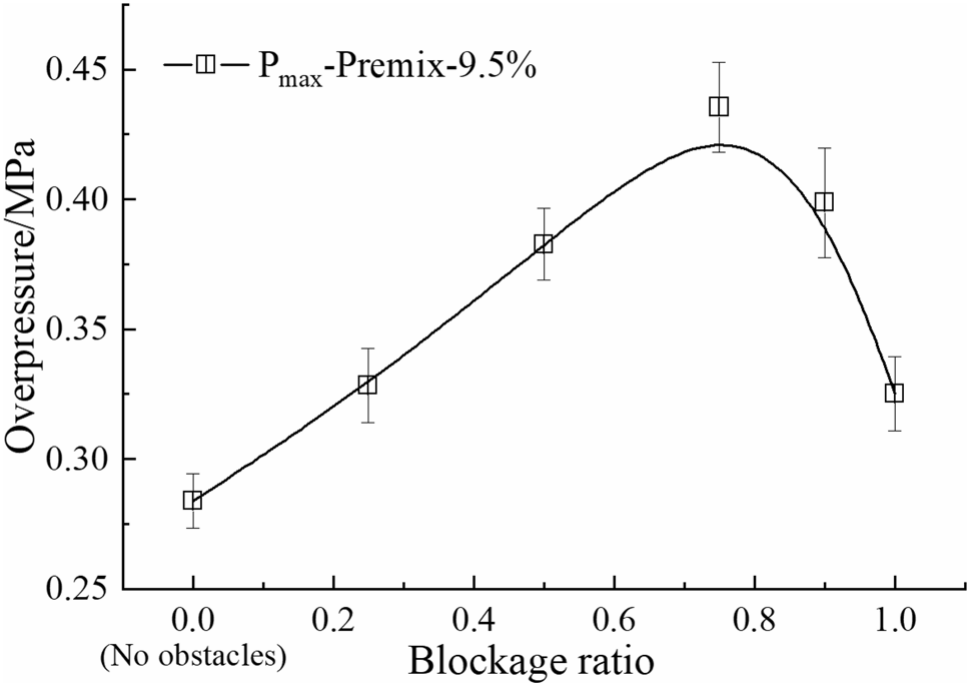
The maximum overpressures as a function of blockage ratio (φ = 1.0, Lb = 0.2 m).

The main experimental parameters are listed in Table 1. According to the mining state and geological conditions at the time of several gas explosion accidents occurrence, five blockage length-diameter ratios (α) of the coal gangue were chosen for the experiment, that is 0.5, 1.0, 2.0, 4.0, and 8.0, corresponding to blockage lengths (L_b_) of 0.1, 0.2, 0.4, 0.8, and 1.6 m, respectively. The blocked length-diameter ratio is the ratio of the axial length (L_b_) of the gangue to the equivalent diameter (d_0_) of the pipeline, which is used to represent the degree of obstruction by the gangue in the axial direction. Previous studies showed that when the blockage ratio of crushed rock is 75% and the accumulation void fraction is 0.55, the maximum overpressure is significantly higher than other conditions^27^, as shown in Fig. 2. Meanwhile, the obstacle located in the center of the premixing area could bring a higher explosion intensity, and it also easier to reflect the differences between the blocking conditions^9^. Therefore, the crushed gangue blockage ratio was set to 75%, the accumulation void fraction was set to 0.55, which was measured by the drainage method.

**Table 1:** Main experimental parameters.

Equivalence ratio (φ)	Blockage length (L_b_)	Blockage length-diameter ratio (α = L_b_/d_0_)	Blockage ratio (BR)	Accumulation void fraction
0.772	0.1 m	0.5	75%	0.55
1.0	0.2 m	1.0		
1.237	0.4 m	2.0		
1.486	0.8 m	4.0		
	1.6 m	8.0

**Figure 3:**
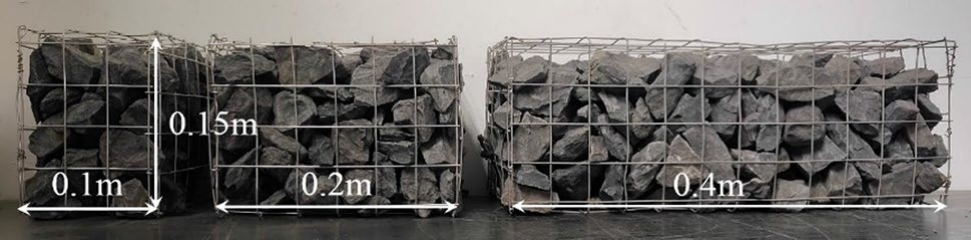
The crushed gangue.

**Figure 4:**
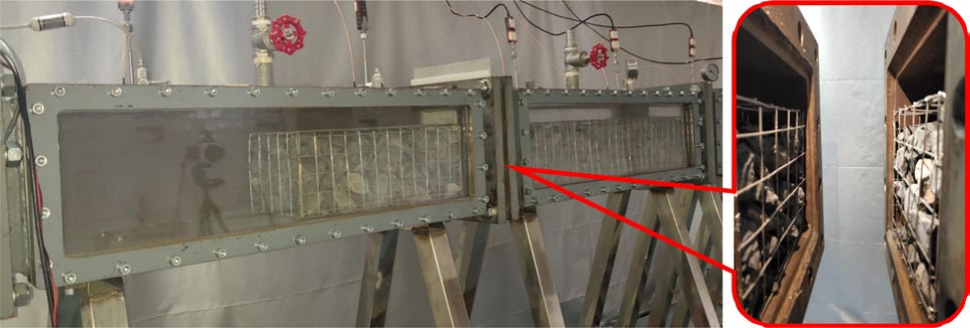
Layout of crushed gangue pass through the polyethylene diaphragm.

The explosion scene in gob was simplified and the position of the crushed gangue from the ignition source was set to 0.5 m, the actual setup is shown in Fig. 3. The gangue used in the experiments have different shapes, we had repeated the experiment 5 times for each condition and found that the results were well reproducible. That is to say, the uniform size (average particle size 35–45 mm) showing a clear similarity in the disturbance on explosion. The axial length of the gangue changes with the length-diameter ratio, when the former exceeds 0.5 m, the gangue passes through chamber 1 and chamber 2. The gangue obstacle is therefore divided into two parts and arranged along the flange to ensure the isolation effect of the polyethylene film and meet the experimental requirements, as shown in Fig. 4. The friction between the chamber and the gangue could keep the gangue stability under the explosion impact cause the gangue self-weight and contact with the chamber inside wall (left side, right side and the bottom).

The premixed gas is prepared by partial pressure method^28,29^. Before the experiment, chamber 1 and chamber 2 were sealed with polyethylene film and an aluminum plate. Chamber1 was then put under vacuum until reaching − 0.095 MPa. The pre-mixed gas was then injected, stabilized to normal pressure, and maintained for 20 min. When the flow field in the chamber stabilized, the methane detector was used to measure the gas concentration to ensure a concentration error of less than ± 0.2%. The aluminum plate was then removed and the igniter was ignited in 10 s (to ensure the accuracy of premixed gas concentration), and a signal was simultaneously sending to trigger the data acquisition instrument and high-speed camera. Upon completion of the experiment, the collected data were saved and the chamber was cleaned. All the experiments were carried out under room temperature and pressure conditions. To ensure the reliability of the results, each experiment was repeated 3 times.

## Results and discussion

### Characteristics of flame morphology and propagation velocity

**Figure 5:**
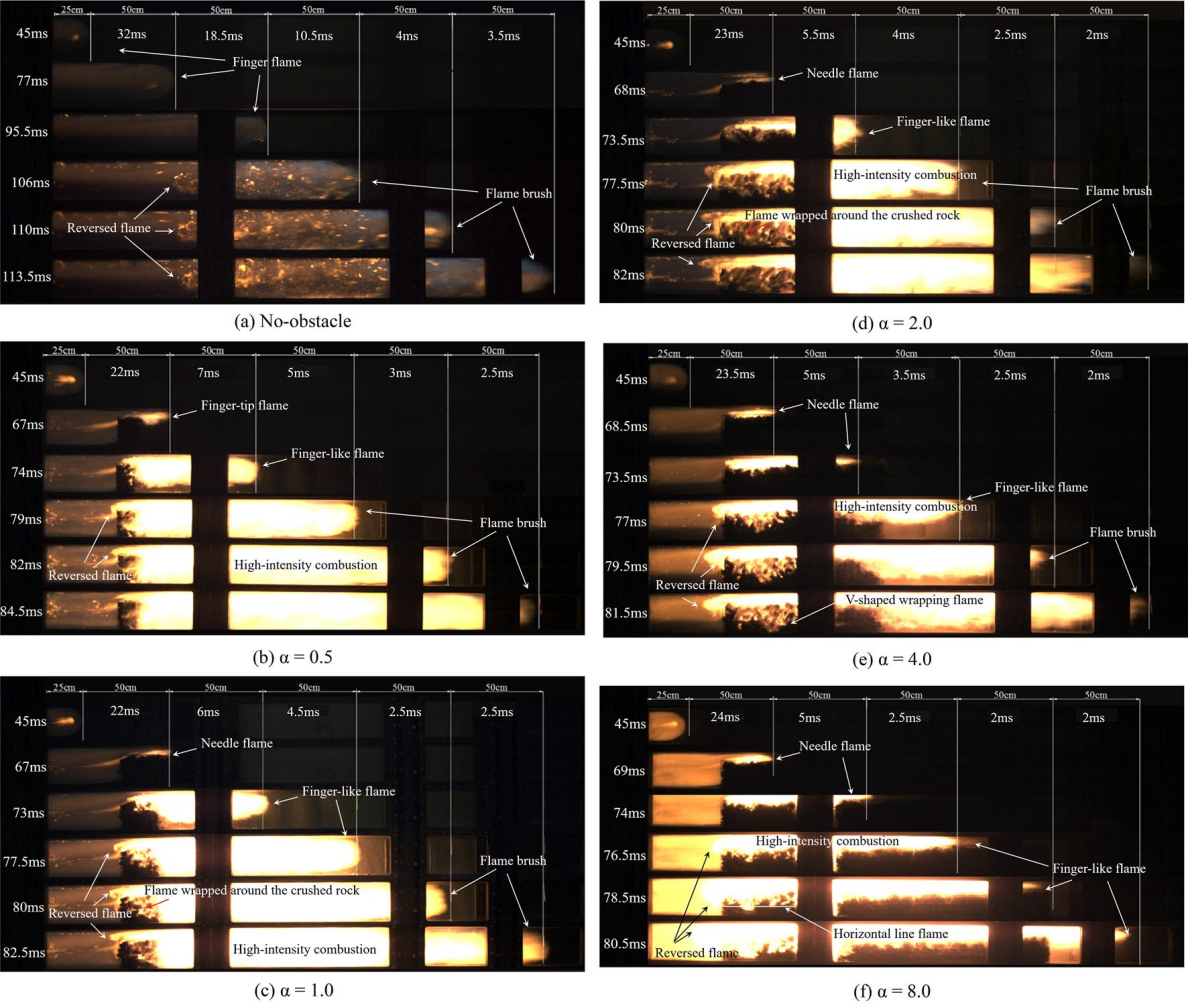
Flame shape of the stoichiometric methane-air explosion under different α.

Figure 5 shows the propagation patterns and corresponding time of the flame front at six different positions under the no-obstacle and five blockage length-diameter ratio (α) conditions using a stoichiometric methane-air ratio. The black part is the chamber structure (edge of the glass window). The white solid lines in Fig. 5 divide the pipeline into six areas, with each area marked the time it takes for the flame to pass through the area. Compared with the no-obstacle case (Fig. 5a), the flame front changes significantly under the blocking effect of crushed gangue. The flame propagation time decreases with the blockage length, and the flame tip propagation time between adjacent positions also decreases with the distance from the ignition.

The propagation process in Fig. 5b–f can be divided into three stages, which include finger propagation, needle propagation, and deformation propagation. The finger propagation stage represents the initial spherical flame until the state immediately before contacting the gangue, in which no obstacle disturbance is present and the flame morphology is dominated by laminar combustion^30^. The needle propagation stage covers the period that the flame passes through the obstructed area, and turbulent combustion is generated by a disturbance of the accumulated material, which is mainly present as bright yellow needle-shaped flames. The distance from the flame front to the pipeline exit after passing through the blocked area is represented by the deformation propagation stage, in which the flame enters the non-blocking space after being disturbed by the crushed gangue and continues to rapidly spread, and a similar phenomenon is found in literature^31^.

During the finger propagation stage, the six flames exhibit the same flame shape. For the non-obstacle condition (Fig. 5a), the flame does not show a typical tulip-shape. This is because the open explosion chamber and there is no severe pressure reflection and superposition during the propagation process^32^. The finger flame is therefore the main form during the entire explosion process in the no-obstacle case^33^.

During the needle propagation phase, the flame fronts exhibit significant differences, as shown in Fig. 5b–f. The length of the needle flame increases with blocking length. The flame brightness increases with the α. Meanwhile, the reversed flame appeared in the upstream of the obstacle during the explosion (Fig. 5c–f), and the reverse propagation distance was positively correlated with α. This indicates that the longer the length of the crushed gangue, the higher the degree of interference to the flame. It is also in good agreement with the research of Sun song et al.^8^.

During the deformation stage, the flame propagates in the direction of the exit, while the flame inside the piled gangue begins to spread from top to bottom. The flame spreads to the interior of the gangue in chamber 1, shown as the V-shaped flame in Fig. 5e. The flames extend downward in an approximate horizontal line while no notable fire in the crushed gangue outside of the premix zone (Fig. 5f). The reason for no notable fire inside the crushed gangue is that the diffusion of unburned gas in the gangue is relatively limited, and the flame propagates mainly through the upper gap.

According to the analysis above, the disturbance of the coal gangue to the flame mainly comes from two aspects. For one thing, the blockage of obstacles reduces the cross section of the flame propagation channels, which leads to the acceleration of the flame propagation; For another, the sharp protrusions on the coal gangue surface increase the turbulence intensity of the flame front, which accelerates the mixing of burned and unburned gas, and this promotion effect increases with the increase of α.

**Figure 6:**
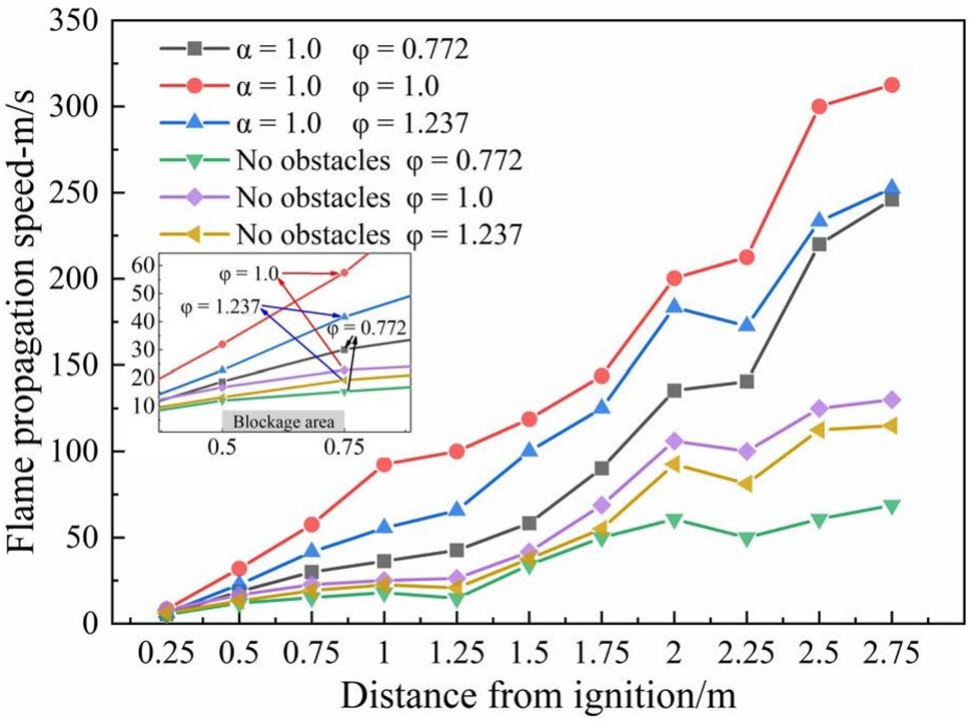
Flame propagation velocity under different equivalence ratios with α = 1.0.

Figure 6 shows the flame propagation velocity under 3 different equivalence ratios with α = 1.0 and with no obstacle condition. Due to the low flame brightness when the equivalence ratio is 1.486, the high-speed camera cannot capture the specific position of the flame front. Therefore, only the flame propagation speeds of equivalence ratio with 0.772, 1.0 and 1.237 are shown in the figure. It can be seen from the figure that the flame propagation velocities under the same blockage condition share a similar trend, and the flame propagation velocity in the pipeline is gradually increasing. The flame propagation velocity under the chemical equivalent concentration is significantly higher than the other two concentration conditions.

**Figure 7:**
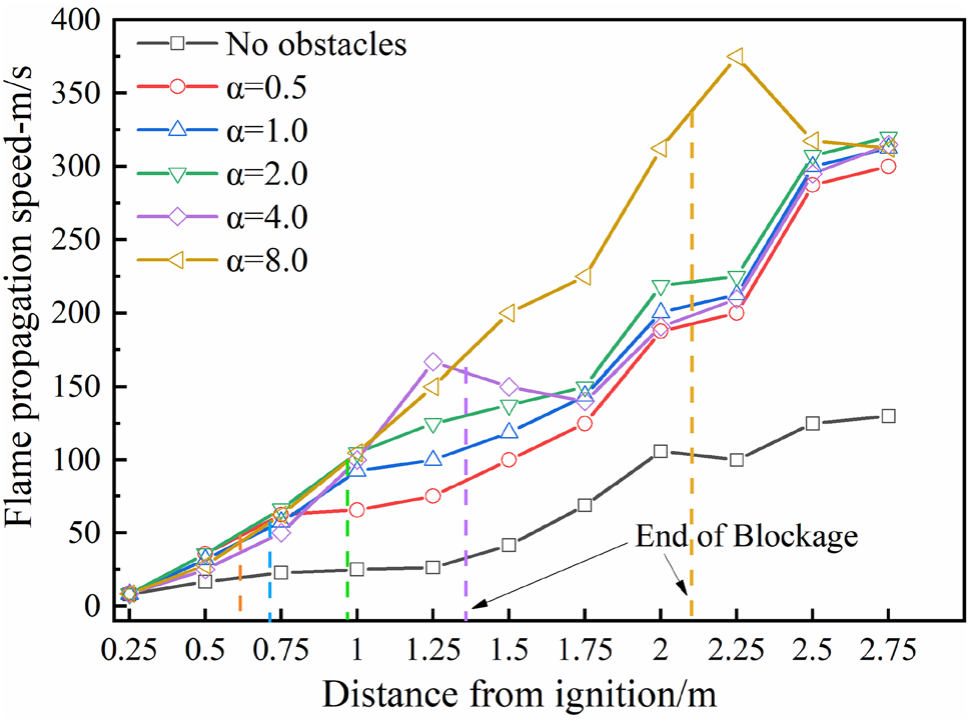
Flame propagation velocity along the pipeline under different α (φ = 1.0).

Figure 7 shows the flame propagation velocity under different blockage length-diameter ratios in the stoichiometric methane-air explosion. The dotted line represents the end blockage position. The flame propagation velocity under the crushed gangue is substantially higher than that in the no-obstacle case. The disturbance of the crushed gangue increases the wrinkle of flame surface and accelerates the flame propagation^12^. The fluctuation phenomenon of the flame propagation velocity along the pipeline under various experimental conditions may be caused by the pulsation effect generated in the explosion^34,35^.

The flame propagation velocity at the end of the blockage area increases with increasing blockage length. At the end of the blockage area disturbance, the increase rate of the flame propagation velocity decreases to a certain extent and the flame propagation velocity changes significantly when α equals 4.0 and 8.0, reaching peak values at 1.25 and 2.25 m, respectively, which then begin to decrease. This is because the flame reaches a larger propagation velocity under the blocking effect and the jet flame front enters a larger static space when the blocking disturbance ends^29,36^, the expansion and acceleration capacity of the combustion products decreases, thus the flame propagation speed decreases to a certain extent. In addition, owing to the continuous combustion of fuel and expansion of products^37^, the flame front gradually increases to a higher velocity, as shown in Fig. 7. For the α of 8.0, the flame speed shows a significant drop, mainly because the end of the blockage is closer to the open end and the flame propagation speed is easily affected by the external atmosphere.

In summary, the crushed gangue significantly accelerates the propagation velocity of methane-air explosion flames. The propagation velocity at the end of the blocked area increases with the α. Longer blockage lengths are associated with more notable obstruction effects and severe disturbances of the flame propagation caused by the irregular upper surface, resulting in the continuous increase of turbulent combustion intensity. Therefore, when the crushed gangue in coalmine gob forms a continuous blocking state, the acceleration effect of this continuous accumulation state may enhance the gas explosion intensity.

### The variation of peak overpressure and peak pressure rise rate

**Figure 8:**
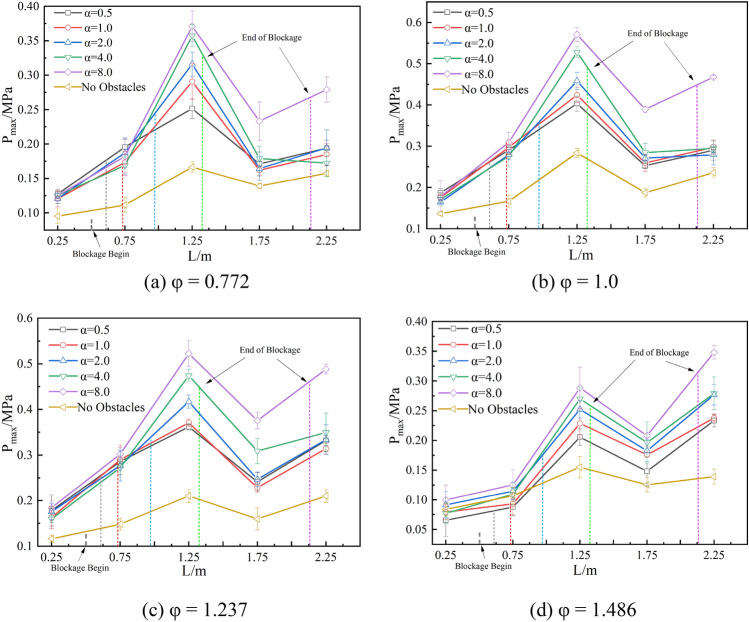
Peak overpressures under different blockage length-diameter ratios.

Figure 8 shows the peak overpressure distribution at each measurement point in the pipeline for the four equivalent ratio methane-air explosion conditions with five blockage lengths. The overpressures in the pipeline present a fluctuating distribution due to the oscillating propagation of the explosion^38^. The peak overpressure of each measurement point in the pipeline is clearly higher under blockage conditions than that of the no-obstacle condition. The maximum peak overpressure of a methane-air explosion was obtained at a blockage length of 1.6 m, corresponding to 0.371 MPa (φ = 0.772), 0.571 MPa (φ = 1.0), 0.523 MPa (φ = 1.237), and 0.348 MPa (φ = 1.486), all of which are considerably higher than the no-obstacle condition of 0.167 MPa (φ = 0.772), 0.284 MPa (φ = 1.0), 0.21 MPa (φ = 1.237), and 0.155 MPa (φ = 1.486).

The maximum peak overpressure distribution of the pipeline under the same equivalence ratio shows a similar trend and both two overpressures increase with the blockage length-diameter ratio. When α = 8.0, the peak overpressure is significantly greater than the other ratios. This is because the gangue blockage reduces the effective cross-sectional area of the pipeline. Under the disturbance of the jet and reflected pressure waves^26^, the flame burning intensity continues to increase, resulting in a relatively high overpressure at measurement point 3. In addition, the explosion intensity increased with disturbance length for φ = 1.486, this also could be a potential risk in coal mines. Therefore, when a gas explosion in the gob is disturbed by coal gangue and spreads to the working area, the blockage length and gas concentration both play an important role in determining the extent of damage.

There is little difference of overpressure between measurement points 1 and 2, while points 3, 4, and 5 are remarkably affected by the obstacles. The overpressure at point 4 is lower than that at point 3 and point 5, which due to the superposition of explosion pressure waves. In addition, when φ = 0.772, 1.0 and 1.237, the various of overpressure at point 4 is similar to that at point 5, and when φ = 1.486, the overpressure at point 4 is similar to that at point 3. Therefore, compared with measurement points 1, 2 and 4, the overpressures at measurement points 3 and 5 are more representative. The overpressures at point 3 represent the maximum value of explosion intensity, and the values at point 5 represent the influence range of explosion. The paper focuses on analyzing the parameters according to the 2 measurement points.

**Figure 9:**
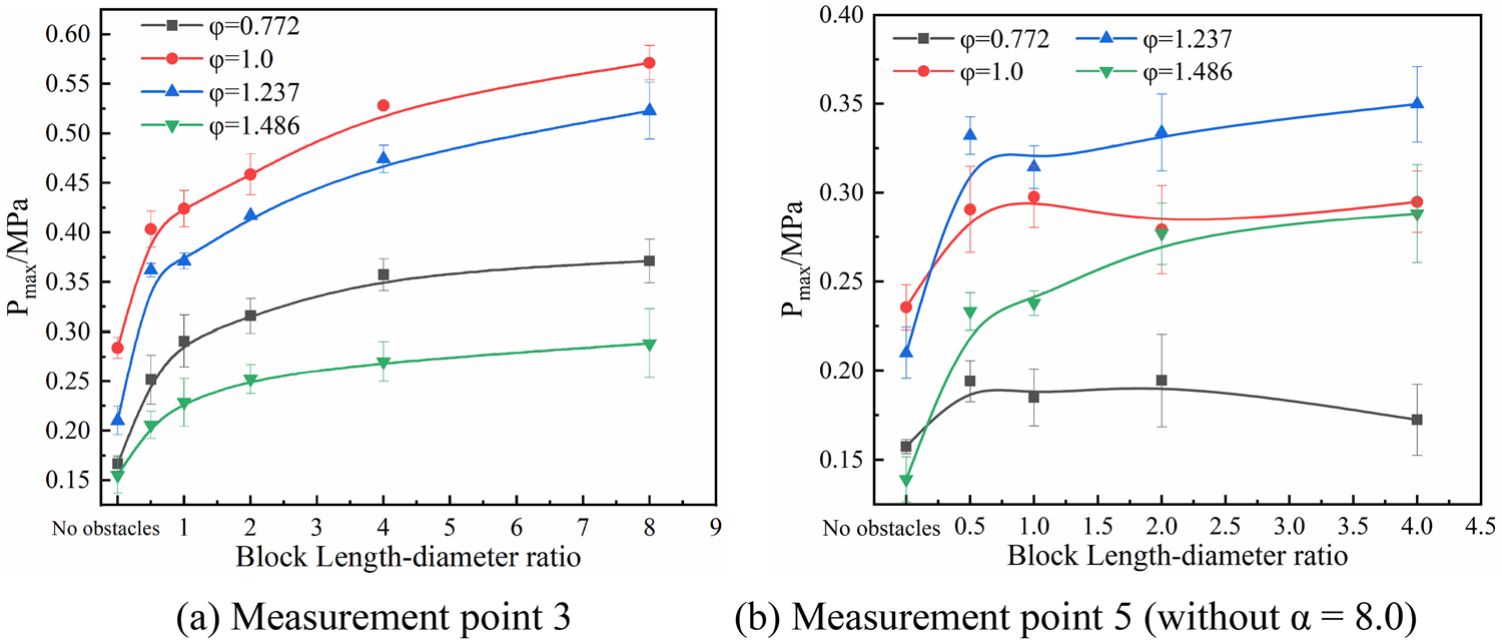
Peak overpressure at measurement points 3 and 5 as a function of α.

Figure 9 shows the peak overpressures as a function of α at measurement points 3 and 5. With the α increases, the overpressures at point 3 all show a gradually increasing tendency (Fig. 9a), and the overpressures at φ = 1.0 are higher than those for other values^39,40^. The overpressure at point 5, on the other hand, shows a different trend. The pressure at measurement point 3 is mainly affected by the combustion rate and the blockage length. The longer the blockage length is, the longer the narrow cross-section propagation channel is formed, and the higher the overpressures are generated. The pressure at measurement point 5 is mainly affected by the combustion rate, the blockage length, and the mixing rate of the premixed gas and air. When the α is 0.5 to 4.0, the overpressures under the conditions of φ = 1.237 and φ = 1.468 increases with the blockage length. However, the overpressures under the conditions of φ = 1.0 does not increase or decrease significantly, for φ = 0.772, the curve presents a downward trend. For the gas explosion under fuel-lean conditions, the combustion rate increases with the length of the blockage, while the attenuation of the pressure wave may also increase.

For Fig. 9b, the peak overpressure reaches a maximum value at φ = 1.237, which is higher than φ = 1.0 condition. This indicates that the disturbance effect of the crushed gangue may accelerate the mixing of gas and air and reduce the concentration. For methane-air with an equivalent ratio of 1.237, a slight decrease of concentration will result in a larger explosion pressure^41,42^, which expands the explosion scope. The mixing rate of unburned gas and air shows a competitive relation with the combustion rate of the initial concentration for fuel-rich (φ = 1.237 and 1.486) methane explosions. When the burning rate is greater than the mixing rate, the explosion intensity does not change significantly. When the mixing rate is greater than the combustion rate, the gas concentration may decrease (close to the stoichiometric concentration) and a higher combustion intensity may form.

**Figure 10:**
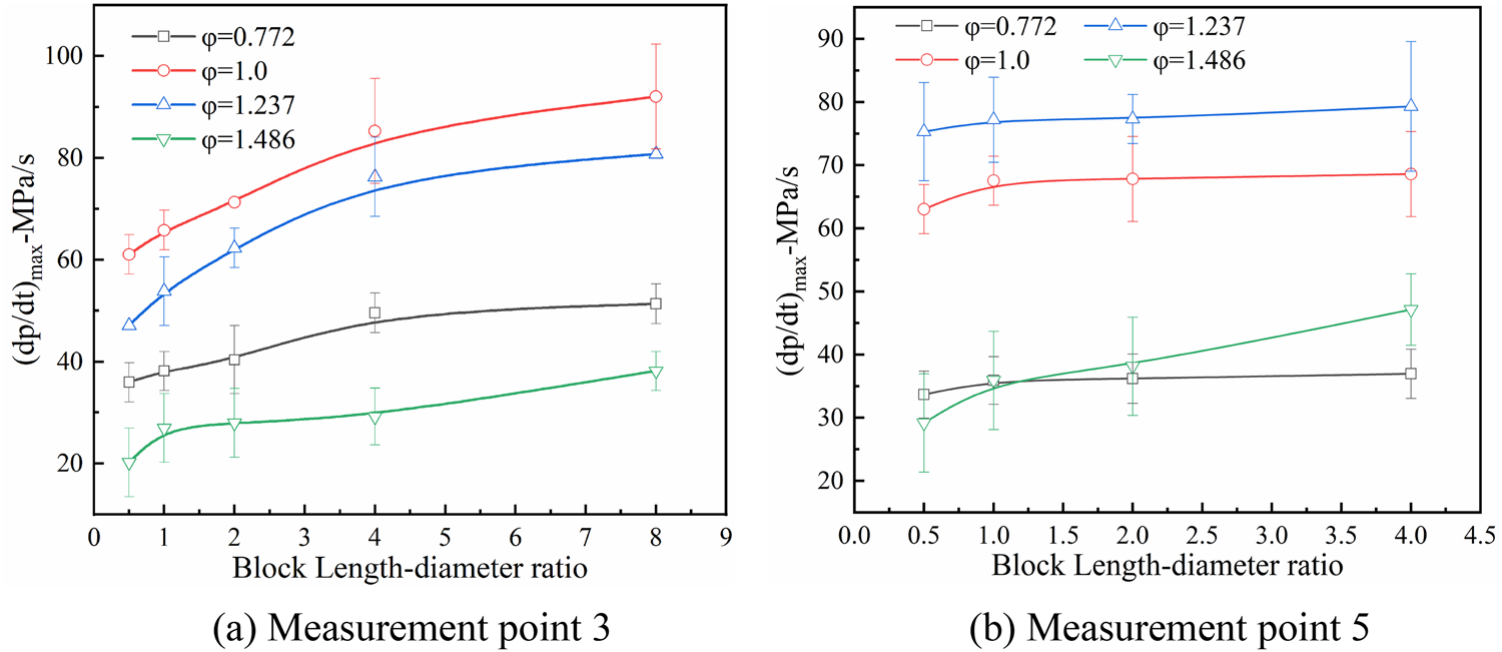
Peak pressure rise rate at measurement points 3 and 5 as a function of α.

Figure 10 shows the peak pressure rise rate at measurement points 3 and 5 as a function of α under the four equivalent ratio conditions. The peak pressure rise rate at measurement point 3 increases with α to the maximum under the stoichiometric concentration condition (Fig. 10a). At measurement point 5, the peak pressure rise rate shows a slight effect from the gangue when α < 4.0 (Fig. 10b). This is because the increased blockage length enhanced the overpressure and flame propagation velocity. As shown in Fig. 10b, the peak pressure rise rate at measurement point 5 reaches the maximum when φ = 1.237, which is consistent with Fig. 9b.

A significant difference in Fig. 10 is when φ = 0.772 and 1.0, the peak pressure rise rate changes slightly at measurement points 3 and 5, but at φ = 1.237 and 1.486, the peak pressure rise rate at measurement point 5 is significantly higher than at measurement point 3. This is because the premixed gas in front of the flame in the pipeline could mix with the air nearby (the disturbance of loose rock also accelerated this mixing), which reduced the premixed gas concentration to a certain extent. When the equivalence ratio is 1.237, the explosion could maintain a higher burning rate after the peak explosion intensity. Therefore, a higher overpressure rise rate is formed at the measurement point 5. When the equivalence ratio is 1.468, due to the low flame propagation velocity, the mixing degree of the premixed gas and air is also low. The burning intensity gradually increases with the flame spreading. Therefore, the maximum pressure is obtained at the measurement point 5.

**Figure 11:**
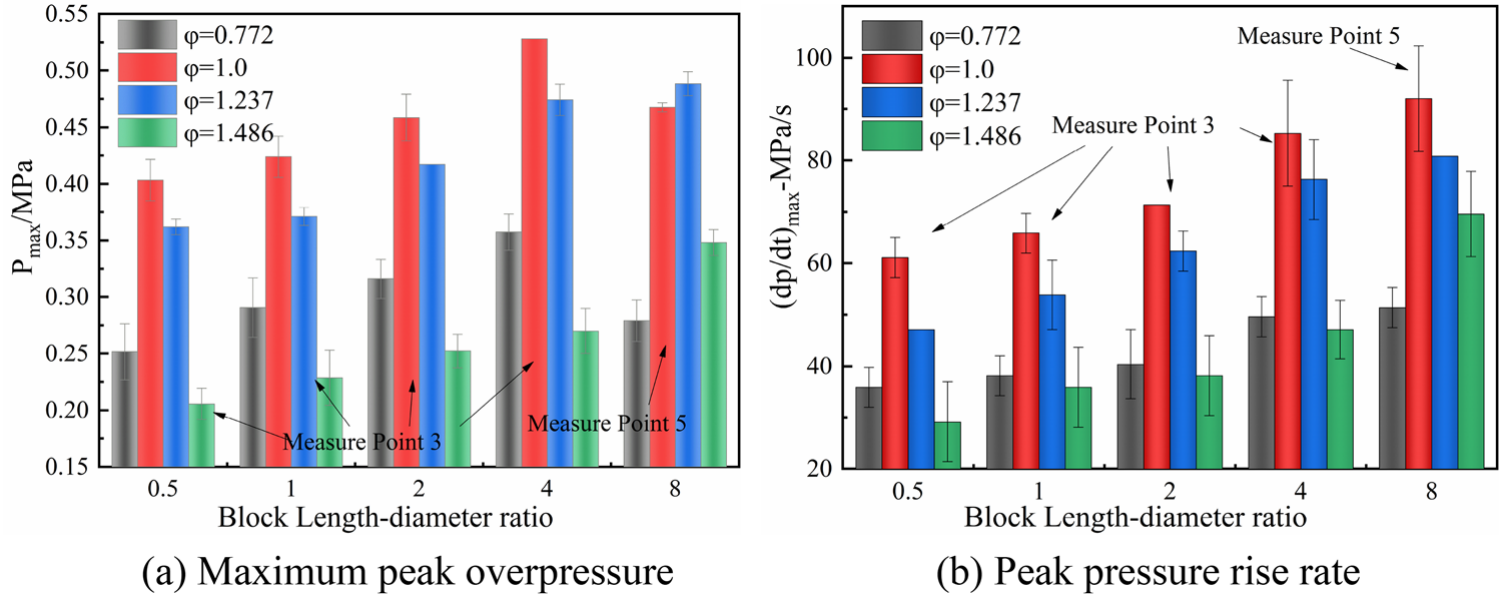
Maximum peak overpressure and peak pressure rise rate in the working area.

According to the experimental and the working area conditions in coal mining, this paper compares the maximum overpressure variation and peak pressure rise rate with different blockage length-diameter ratios, as shown in Fig. 11. When α = 0.5, 1.0, and 2.0, the maximum peak overpressure outside of the obstruction area is obtained at measurement point 3 (Fig. 8). For α = 4.0, the end of the blockage area is near point 3, thus the maximum peak overpressure outside of the blocked area is counted by point 3. For α = 8.0, the end of the blockage area is near measurement point 5, thus the maximum peak overpressure outside of the blocked area is counted by point 5.

Figure 11a and b show that the α and φ have a significant impact on the explosion intensity outside of the blockage area. When φ = 0.772 and 1.0, the explosive intensity first increases with α and then decreases; when φ = 1.237 and 1.486, the explosive intensity continues to increase with increasing α. And the gas explosion in large-sized roadway is more harmful^43,44^. Therefore, during the mining process, the blockage state of gangue will inevitably induce the acceleration of gas flames, which may lead to a more devastating catastrophe.

### Peak overpressure ratio varies with the blockage length-diameter ratio

**Figure 12:**
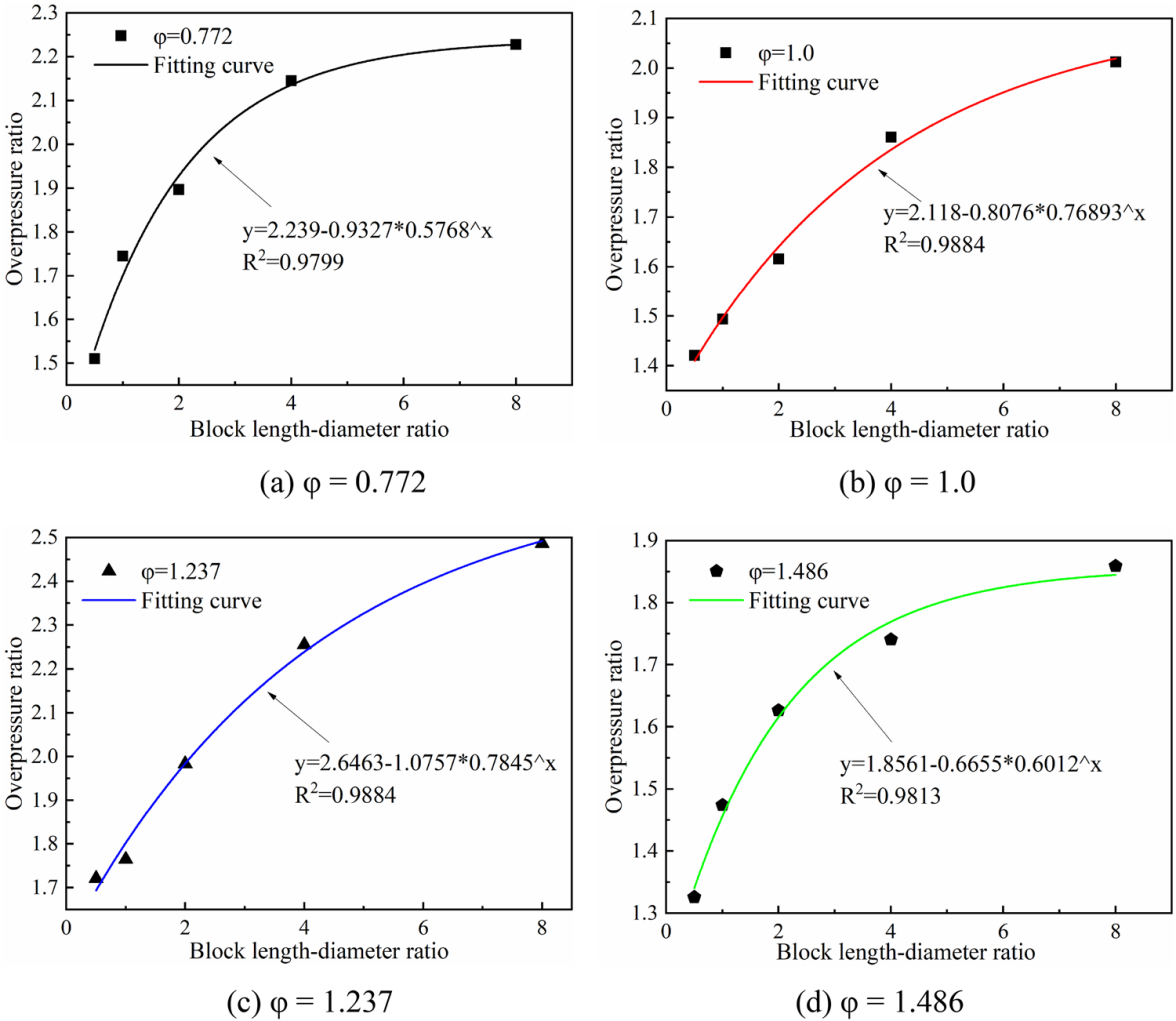
Fitting of overpressure ratio and block length-diameter ratio.

An important aim is to further understand how explosion overpressure characteristics vary with the blockage length of crushed gangue under different equivalence ratios. This paper calculates the peak overpressure ratio, which is the ratio of the maximum peak overpressure (P_max_) under different blockage length-diameter ratios and the maximum peak overpressure (P_Nmax_) under no-obstacles condition, expressed as σ_p_ = P_max_/P_Nmax_ (Table 2). Fitting of the overpressure ratio σ_p_ with the α shows a strong negative exponential function relationship, as shown in Fig. 12.

**Table 2:** Peak overpressure ratios under various explosion conditions.

Equivalence ratio (φ)	Blockage length-diameter ratio (α)				
	0.5	1.0	2.0	4.0	8.0
0.772	1.51	1.745	1.896	2.145	2.227
1.0	1.421	1.494	1.615	1.861	2.012
1.237	1.721	1.765	1.984	2.255	2.486
1.486	1.326	1.474	1.627	1.741	1.859

Under the four equivalent ratios, the increase of the overpressure ratio gradually tends to flatten with increasing blockage length-diameter ratio. The explosion intensity increases with block length, but the increment gradually decreases. When α reaches 8.0, for φ = 1.0 and 1.237, the explosive overpressure shows a significant increase trend, whereas for φ = 0.772 and 1.486, the increase rate notably weakens. This indicates that when φ = 1.0 and 1.237, the explosion is more sensitive to the crushed gangue blockage length and reaches a higher intensity with increasing blockage length.

The negative exponential function relationship reflects the influence of continuous disturbance of crushed gangue on the explosion strength. The blockage length determines the degree of continuous disturbance and the gas concentration determines the rate of reaction. The gangue induces the gas explosion flame to accelerate combustion, and the accelerated combustion of the flame front also means the accelerated depletion of fuel. With the depletion of premixture, the increase of peak overpressure gradually decreases and the maximum overpressure may tend to a certain value.

## Conclusions

This paper experimentally studied the influence of blockage length-diameter ratio (α = 0.5 ~ 8.0) of crushed gangue on the propagation characteristics of gas explosion. It was found that the parameters such as flame propagation velocity, explosion overpressure and pressure rise rate changed significantly with α. The main conclusions were as follows:

1. The degree of disturbance of the flame front increases with the increase of blockage length-diameter ratios (0.5 ~ 8.0), and the brightness increases significantly. The bright white flame and the reverse propagation flame appears when α ≥ 1.0, The propagation distance of reversed flame is positively correlated with the blockage length-diameter ratio, and reaches the maximum when α = 8.0.
2. The disturbance intensity of methane-air explosion flames by crushed gangue increases with the blockage length-diameter ratio. For 0.5 ≤ α ≤ 8.0, the flame propagation velocity at the end of the blockage area increases with the obstruction length. Under the stoichiometric ratio, the maximum peak flame velocity is 2.95 times of that under no-obstacle conditions.
3. The explosion intensity of the four equivalent ratios increases with the blockage length-diameter ratio (0.5 ~ 8.0). For α = 8.0, the maximum overpressure at each equivalent ratio is 0.371 MPa (φ = 0.772), 0.571 MPa (φ = 1.0), 0.523 MPa (φ = 1.237) and 0.348 MPa (φ = 1.486), respectively.
4. The overpressure ratios (σ_p_) show a negative exponential distribution with α under the four equivalence ratios. Explosions with the equivalent ratio equals 1.0 and 1.237 is more sensitive to the crushed gangue blockage length. It is necessary to avoid the formation of such a state of promoting blockage state in a coal mine gob.

## Data Availability

The datasets used and analysed during the current study are available from the corresponding author on reasonable request.

## List of symbols

BR: Blockage ratio
L_b_: The axial length of the crushed gangue, m
d_0_: Equivalent diameter of the pipeline, m
P_max_: Peak overpressure, MPa
P_*N*__max_: The maximum peak overpressure under no-obstacles condition, MPa
(dp/dt)_max_: Peak pressure rise rate, MPa/s

## Greek symbols

α: The blockage length-diameter ratio, α = L_b_/d_0_
σ_p_: The ratio of the maximum peak overpressure under different blockage length-diameter ratios and the maximum peak overpressure under no-obstacles condition, σ_p_ = P_max_/P_*N*__max_
φ: Equivalence ratio

## References

[1] Lin B, Zhou S, Zhang R (1999). The influence of obstacles on the flame and explosion wave in the process of gas explosion. J. China Univ. Min. Technol..

[2] Kundu S, Zanganeh J, Moghtaderi B (2016). A review on understanding explosions from methane-air mixture. J. Loss Prev. Process Ind..

[3] Wang L, Si R, Li R, Huo Y (2018). Experimental investigation of the propagation of deflagration flames in a horizontal underground channel containing obstacles. Tunn. Undergr. Sp. Technol..

[4] Wang LQ, Ma HH, Shen ZW, Chen DG (2019). Effect of a single orifice plate on methane-air explosion in a constant volume vessel: Position and blockage ratio dependence. Exp. Therm. Fluid Sci..

[5] Xu Y, Huang YM, Ma GW (2020). A review on effects of different factors on gas explosions in underground structures. Undergr. Sp..

[6] Wang J, Liu G, Zheng L, Pan R, Lu C, Wang Y, Fan Z, Zhao Y (2022). Effect of opening blockage ratio on the characteristics of methane/air explosion suppressed by porous media. Process Saf. Environ. Prot..

[7] Li G, Du Y, Liang J (2018). Characteristics of gasoline-air mixture explosions with different obstacle configurations. J. Energy Inst..

[8] Sun S, Qiu Y, Xing H, Wang M (2020). Effects of concentration and initial turbulence on the vented explosion characteristics of methane-air mixtures. Fuel.

[9] Li G, Wu J, Wang S, Bai J, Wu D, Qi S (2021). Effects of gas concentration and obstacle location on overpressure and flame propagation characteristics of hydrocarbon fuel-air explosion in a semi-confined pipe. Fuel.

[10] Hou Z, Wang D, Zhang W (2023). Study on effect of transverse concentration gradients in pipeline on methane explosion characteristics. Int. J. Hydrogen Energy.

[11] Brune JF, Grubb JW, Bogin GE, Marts JA, Saki SA (2015). In Lessons learned from research about methane explosive gas zones in coal mine gobs. SME Annu. Meet. Feb..

[12] Fig, M.; Bogin, G.; Brune, J.; Strebinger, C. The impact of rock pile location on the propagation of methane flames in simulated and experimental flame reactors, 2018 SME Annual Conference and Expo and 91st Annual Meeting of the SME-MN, United states, 2018; p Hexagon Mining.

[13] Luo Z, Zhang F, Liu L (2024). Effects of mixing CO and H_2_ as main components on the explosion characteristics and dynamics properties of methane. Int. J. Hydrogen Energy.

[14] Yu M, Zheng K, Chu T (2016). Gas explosion flame propagation over various hollow-square obstacles. J. Natl. Gas Sci. Eng..

[15] Na'inna AM, Phylaktou HN, Andrews GE (2017). Explosion flame acceleration over obstacles: Effects of separation distance for a range of scales. Process Saf. Environ. Prot..

[16] Zhang Q, Wang Y, Lian Z (2017). Explosion hazards of LPG-air mixtures in vented enclosure with obstacles. J. Hazard. Mater..

[17] Wang Q, Liu SH, Shu CM, Ding YB, Li ZM (2017). Influence of different types of obstacles on the propagation of premixed methane-air flames in a half-open tube. Energies.

[18] Di Sarli V, Di Benedetto A, Russo G (2009). Using Large Eddy Simulation for understanding vented gas explosions in the presence of obstacles. J. Hazard. Mater..

[19] Morsy ME, Yang J (2022). The instability of laminar methane/hydrogen/air flames: Correlation between small and large-scale explosions. Int. J. Hydrogen Energy.

[20] Li Y, Yang S, Bi M (2023). Evaluation of hydrogen-blended methane explosion. Int. J. Hydrogen Energy.

[21] Luo Z, Zhou S, Wang T (2023). The weakening effect of the inhibition of CO2 on the explosion of HCNG with the increase of hydrogen: Experimental and chemical kinetic research. Int. J. Hydrogen Energy.

[22] Liu L, Luo Z, Eckart S (2023). Chunyan Zhang, Investigation of _C_2_H_6, _C_2_H_4, CO and _H_2 on the explosion pressure behavior of methane/blended fuels. Int. J. Hydrogen Energy.

[23] Liu Z, Zhong X, Zhong Q (2023). Study on acceleration and suppression properties of coal gangue with different void fractions on gas explosion propagation. J. Loss Prev. Process Ind..

[24] Zhou S, Gao J, Luo Z (2021). Effects of mesh aluminium alloy and aluminium velvet on the explosion of H_2_/air, CH_4_/air and C_2_H_2_/air mixtures. Int. J. Hydrogen Energy.

[25] Ciccarelli G, Johansen C, Parravani M (2011). Transition in the propagation mechanism during flame acceleration in porous media. Proc. Combust. Inst..

[26] Ciccarelli G, Johansen C, Kellenberger M (2013). High-speed flames and DDT in very rough-walled channels. Combust. Flame.

[27] Zhenqi L (2021). Study on the Effect of Crushed Rock on Propagation Characteristics and Suppression of Gas Explosion.

[28] Yu M, Yang X, Zheng K, Zheng L, Wan S (2018). Experimental study of premixed syngas/air flame propagation in a half-open duct. Fuel.

[29] Wang J, Fan Z, Wu Y, Zheng L, Pan R, Wang Y (2021). Effect of abrupt changes in the cross-sectional area of a pipe on flame propagation characteristics of CH_4_/Air mixtures. ACS Omega.

[30] Zheng L, Dou Z, Du D (2019). Study on explosion characteristics of premixed hydrogen/biogas/air mixture in a duct. Int. J. Hydrogen Energy.

[31] Duan Y, Long F, Huang J (2022). Effects of porous materials with different thickness and obstacle layout on methane/hydrogen mixture explosion with low hydrogen ratio. Int. J. Hydrogen Energy.

[32] Jin K, Duan Q, Liew KM, Peng Z, Gong L, Sun J (2017). Experimental study on a comparison of typical premixed combustible gas-air flame propagation in a horizontal rectangular closed duct. J. Hazard. Mater..

[33] Luo Z, Hao Q, Wang T, Li R, Cheng F, Deng J (2020). Experimental study on the deflagration characteristics of methane-ethane mixtures in a closed duct. Fuel.

[34] Wang Q, Shen Z, Guo Z, Ma H, Wu H (2013). Analysis on propagation characteristics of premixed methane-air flame in an half-open tube based on high-speed video camera. Explos. Mater..

[35] Lv C, Wu Z (2017). Flame thickness and propagation characteristics of premixed methane-air explosion with a small filling ratio in an open-ended steel pipe. Appl. Therm. Eng..

[36] Wang J, Wu Y, Zheng L, Yu M, Pan R, Shan W (2020). Study on the propagation characteristics of hydrogen/methane/air premixed flames in variable cross-section ducts. Process Saf. Environ. Prot..

[37] Han S, Yu M, Yang X, Wang X (2020). Effects of obstacle position and hydrogen volume fraction on premixed syngas-air flame acceleration. Int. J. Hydrogen Energy.

[38] Wang L-Q, Ma H-H, Shen Z-W, Pan J (2021). A comparative study of the explosion behaviors of H-2 and C2H4 with air, N2O and O-2. Fire Saf. J..

[39] Niu Y, Shi B, Jiang B (2019). Experimental study of overpressure evolution laws and flame propagation characteristics after methane explosion in transversal pipe networks. Appl. Therm. Eng..

[40] Yang J, Guo J, Wang C, Wang X, Li J, Zhang S, Duan Z, Yang F (2020). Effect of equivalence ratio on hydrogen-methane-air deflagration in a duct with an open end. Fuel.

[41] Ma Q, Zhang Q, Li D, Chen J, Ren S, Shen S (2015). Effects of premixed methane concentration on distribution of flame region and hazard effects in a tube and a tunnel gas explosion. J. Loss Prev. Process Ind..

[42] Liu Z, Zhong X, Zhang Q, Lu C (2020). Experimental study on using water mist containing potassium compounds to suppress methane/air explosions. J. Hazard. Mater..

[43] Tan B, Liu Y, Liu H, Wang H, Li T (2021). Research on size effect of gas explosion in the roadway. Tunn. Undergr. Sp. Technol..

[44] Zhu Y, Wang D, Shao Z, Xu C, Li M, Zhang Y (2021). Characteristics of methane-air explosions in large-scale tunnels with different structures. Tunn. Undergr. Sp. Technol..

